# Variation Analysis in Premenopausal and Postmenopausal Breast Cancer Cases

**DOI:** 10.3390/jpm14040434

**Published:** 2024-04-20

**Authors:** Ibrahim Halil Erdogdu, Seda Orenay-Boyacioglu, Olcay Boyacioglu, Duygu Gurel, Nurten Akdeniz, Ibrahim Meteoglu

**Affiliations:** 1 Department of Molecular Pathology, Faculty of Medicine, Aydin Adnan Menderes University, 09010 Aydin, Turkey; ibrahim.halil.erdogdu@adu.edu.tr (I.H.E.); imeteoglu@adu.edu.tr (I.M.); 2 Department of Medical Genetics, Faculty of Medicine, Aydin Adnan Menderes University, 09010 Aydin, Turkey; 3 Faculty of Engineering, Aydin Adnan Menderes University, 09010 Aydin, Turkey; oboyaci@adu.edu.tr; 4 Department of Pathology, Faculty of Medicine, Dokuz Eylul University, 35220 Izmir, Turkey; duygu.gurel@deu.edu.tr; 5 Private Obstetrics & Gynecology, and Infertility Clinic, 35050 Izmir, Turkey; nakdeniz21@gmail.com

**Keywords:** breast cancer, molecular subtyping, NGS, gene variations

## Abstract

Menopausal status affects the prognoses and consequences of breast cancer. Therefore, this retrospective study aimed to reveal the molecular variation profile differences in breast cancer patients according to their menopausal status, with the hypothesis that the molecular variation profiles will be different at premenopausal and postmenopausal ages. Breast cancer patients (*n* = 254) who underwent molecular subtyping and QIAseq Human Breast Cancer NGS Panel screening between 2018 and 2022 were evaluated retrospectively. Their menopausal status was defined by age, and those aged 50 years and above were considered postmenopausal. Of the subjects, 58.66% (*n* = 149) were premenopausal and 41.34% (*n* = 105) were postmenopausal. The mean age at the time of diagnosis for all patients was 49.31 ± 11.19 years, with respective values of 42.11 ± 5.51 and 59.54 ± 9.01 years for the premenopausal and postmenopausal groups, respectively (*p* = 0.000). Among premenopausal patients, the percentages of patients in BCa subtypes (luminal A, luminal B-HER2(−), luminal B-HER2(+), HER2 positive, and triple-negative) were determined to be 34.90%, 8.05%, 26.17%, 10.74%, and 20.13%, respectively, while in the postmenopausal group, these values were 39.05%, 16.19%, 24.76%, 6.67%, and 13.33%, respectively (*p* > 0.05). Considering menopausal status, the distribution of hormone receptors in premenopausal patients was ER(+)/PgR(+) 63.76%, ER(−)/PgR(−) 23.49%, ER(+)/PgR(−) 10.74%, and ER(−)/PgR(+) 2.01%, respectively, while in postmenopausal women, this distribution was observed to be 74.29%, 23.81%, 1.90% and 0.00% in the same order (*p* = 0.008). The most frequently mutated gene was *TP53* in 130 patients (51.18%), followed by *PIK3CA* in 85 patients (33.46%), *BRCA2* and *NF1* in 56 patients (22.05%), *PTEN* in 54 patients (21.26%), and *ATR* and *CHEK2* in 53 patients (20.87%). *TP53, PIK3CA, NF1, BRCA2, PTEN*, and *CHEK2* mutations were more frequently observed in premenopausal patients, while *TP53, PIK3CA, BRCA2, BRCA1,* and *ATR* mutations in postmenopausal patients. These findings contribute to a deeper understanding of the underlying causes of breast cancer with respect to menopausal status. This study is the first from Turkey that reflects the molecular subtyping and somatic mutation profiles of breast cancer patients according to menopausal status.

## 1. Introduction

Breast cancer (BCa) is the most common neoplasia among women worldwide, accounting for approximately 15% of new cancer cases and 7% of cancer deaths in 2023 [1]. According to GLOBOCAN data, a 46% increase is expected in BCa cases by 2040 [2]. Risk factors of BCa could be related to genetic and epigenetic, and behavioral and non-behavioral factors [3]. Menopause is also an important risk factor as BCa has different molecular characteristics and reasons in premenopausal and postmenopausal women. For instance, being overweight is a risk factor for BCa in postmenopausal women; however, it is less associated with premenopausal BCa, though some studies suggest an inverse relationship [1,2]. Molecular subtypes of BCa exhibit variations in risk factors, such as genetic predisposition, therapeutic strategies, and prognosis. Additionally, they present distinct age-incidence profiles, particularly during menopause. From a public health and patient perspective, the population sizes of women at risk of BCa, both premenopausal and postmenopausal, vary significantly among countries. This variation is influenced by the demographic composition (sex, ethnic origin, etc.) of each country’s population, and particularly by the age distribution of population. Relatively higher breast density results in the late diagnosis of premenopausal BCa. Eventually, the outcomes of BCa in women vary between younger and older patients, indicating differences in disease progression, treatment response, and overall prognosis based on age [3,4]. Therefore, studying the burden of BCa and its molecular gene profile based on menopausal status holds significant importance in guiding BCa prevention and detection efforts, as well as healthcare planning [3]. Therefore, in this retrospective study with a cohort of Turkish BCa patients, the aim was to reveal the differences of somatic gene variant profiles and disease molecular subtypes according to menopausal status.

## 2. Materials and Methods

### 2.1. Ethical Approval and Patients

The study was approved by the Institutional Non-Interventional Clinical Research Ethics Committee (#2023/172). The Helsinki Declaration criteria were taken into consideration.

In this study, which included a retrospective analysis of a database prepared with prospective data entry, the medical records of all the patients diagnosed with BCa and followed up at the Oncology Department of Adnan Menderes University Medical Faculty Hospital and referred to the Molecular Pathology Laboratory between 2018 and 2022 were retrospectively reviewed. Patients without data on estrogen receptor (ER), progesterone receptor (PgR), human epidermal growth factor receptor 2 (HER2) statuses, proliferative cell nuclear antigen (Ki-67) index, or next generation sequencing (NGS) breast cancer panel results were excluded. The analyses were conducted on data from a total of 254 patients. Demographic, pathological, and molecular characteristics, including age, menopausal status, tumor histopathology, ER, PgR, HER2 status, Ki-67 index data, and somatic mutation results, were analyzed for each patient. Menopausal status was defined using age, and patients were grouped into a premenopausal adult group (aged 18–49) and a postmenopausal group (aged 50 and above).

### 2.2. Immunohistochemical Staining

Hormone receptors were determined according to ASCO/CAP guidelines [5]. To prepare the BCa tissues for analysis, they were initially fixed in 10% formaldehyde for 24 h, followed by routine dehydration, clearing, and embedding in paraffin. The tissues were then sliced into continuous sections of 5 μm thickness. Subsequently, the sections were heated at 65 °C, dewaxed using xylene, hydrated using gradient ethanol, and treated with 3% H_2_O_2_ for 10 min at 37 °C to inactivate endogenous peroxidase. Next, antigen retrieval was performed by microwave heating, followed by blocking by normal goat serum. The tissue sections were then incubated at 4 °C overnight using primary antibodies targeting ER, PgR, HER2, and Ki-67. The following day, biotin-labeled secondary antibodies were applied for 30 min at room temperature. The development of the sections was achieved using diaminobenzidine, followed by counterstaining with hematoxylin. The sections were then differentiated using hydrochloric acid ethanol, dehydrated by gradient ethanol, and cleared by xylene before being mounted using neutral gum. Finally, the stained sections were examined microscopically. The negative control for the primary antibody was phosphate-buffered saline. The indication of nuclear staining over 1% of tumor cells was considered as positive staining for ER and PgR. HER2 status was classified according to Dako criteria (Glostrup) as 0, 1+, 2+, or 3+, with scores of 0 and 1+ being considered as negative. The presence of 14% or more tumor cells for Ki-67 marker index was considered highly expressed after nuclear staining. Patients with an HER2 immunohistochemistry score of 3+ were included, while cases with an HER2 status of 1+ or 2+ underwent fluorescence in situ hybridization (FISH) analysis for *HER2* gene amplification.

### 2.3. FISH Analysis

The FISH analysis was performed with an *HER2* dual-color probe kit (Dako A/S, Glostrup, Denmark). The kit contains two fluorescent-labeled probes specific to the *HER2* gene locus (17q11) and *CEP17*. The 4 µm thick sections were obtained from paraffin blocks and transferred onto positively-charged slides, followed by deparaffinization. Subsequently, they were dehydrated in alcohol, and treated with sodium thiocyanate and protease solution. The sections were dehydrated in 70%, 80%, and 100% alcohol and air-dried. The probe kit was denatured at 80 °C for 5 min and then applied, followed by incubation in a humid environment for 12 h. After incubation, post-hybridization washes were performed at room temperature with sodium saline citrate solution. The preparations were washed with buffer solution after hybridization, air-dried in the dark, and covered with a coverslip using DAPI. Subsequently, the *HER2*/neu signals were counted in at least 20 cells for both signals using fluorescence microscopy (Olympus BX51, Tokyo, Japan) at 1000× magnification with immersion oil, employing DAPI, FITC, and TRITC dual and triple filters. The results were calculated according to the ASCO guidelines using the *HER2*/neu signal (red) to *CEP17* signal (green) ratio and the number of *HER2*/neu copies. Cases with a ratio of 2 or more and a copy number of 4 or more were accepted as positive result for *HER2*/neu gene amplification (Figure S1).

### 2.4. Molecular Classification

The tumor immunohistopathological subtypes were categorized following the guidelines [6]. The histopathological subgroups were defined as Luminal A, if ER(+) and/or PgR(+), HER2(−), Ki67 < 20%; Luminal B-HER2(−) if ER(+) and/or PgR(+), HER2(−), and Ki-67 ≥ 20%; Luminal B-HER2(+) if ER(+) and/or PgR(+), and HER2(+); HER2 positive if ER(−), PgR(−), and HER2(+); and Triple negative if ER(−), PgR(−), and HER2(−).

### 2.5. Targeting NGS Panel Analysis

The DNA samples from formalin-fixed paraffin embedded tissues of the patients were extracted using a commercial DNA isolation kit (GeneRead TM FFPE kit, Qiagen, Hilden, Germany). The quality and concentration of the DNA samples were evaluated spectrophotometrically and those with the OD260/OD280 values between 1.8~2.0 were included in the NGS study. Sequencing was performed on an Illumina MiSeq platform (Illumina Inc., San Diego, CA, USA). In this study, the QIAseq Human Breast Cancer Panel (DHS-001Z, Qiagen, Hilden, Germany) containing 93 genes and 4831 primers was utilized. The QIAseq Targeted DNA panels for BCa include full exonic regions of genes plus 10 bases to cover the intron and exon junctions coding for 93 genes (Table 1). For the analysis of the obtained data, the Qiagen Clinical Insight Interpret 8.1.202021 (QCI™) (Qiagen, Hilden, Germany) was used. In addition to the exonic regions of the investigated genes, 20 base pairs in the intron regions at the exon-intron boundaries were also evaluated. The variants detected in the study were classified according to the criteria outlined in the American College of Medical Genetics and Genomics (ACMG) guidelines [7].

### 2.6. Statistical Analysis

The IBM SPSS 25 software (IBM Corp., Armonk, NY, USA) was employed to conduct statistical analysis. A significance level of *p* ≤ 0.05 was considered statistically significant for all analyses. A descriptive analysis was performed, with categorical variables defined as frequencies. The Spearman correlation test was employed for correlation analyses. The percentages and distributions of quantitative variables were presented as median, minimum, and maximum values. Categorical variables were evaluated using chi-square or Fisher’s exact test.

## 3. Results

### 3.1. Patient Characteristics

Of the 254 patients comprising the study population, 149 (58.66%) were premenopausal, and 105 (41.34%) were postmenopausal. The mean age and standard deviation at the time of diagnosis for all patients were 49.31 ± 11.19 years, with respective values of 42.11 ± 5.51 and 59.54 ± 9.01 years for the premenopausal and postmenopausal groups, respectively (*p* = 0.000).

### 3.2. Evaluation of Tumors by Histological Subtypes

When the tumors were evaluated by histological subtype, 85.04% were determined to be invasive ductal carcinoma, 10.24% were invasive lobular carcinoma, and 4.72% were other special types of carcinomas. When evaluated according to menopausal status, 86.58% of premenopausal BCa patients were classified as invasive ductal carcinoma, 10.74% as invasive lobular carcinoma, and 2.68% as other special types of carcinomas. In postmenopausal patients, 82.86% were classified as invasive ductal carcinoma, 9.52% as invasive lobular carcinoma, and 7.62% as other special types of carcinomas. The distribution of ER and PgR in all cases was as follows: ER(+)/PgR(+) 68.11%, ER(−)/PgR(−) 23.62%, ER(+)/PgR(−) 7.09%, and ER(−)/PgR(+) 1.81%. When the relationships between hormone receptors were evaluated, a positive correlation was found between ER and PgR (*p* < 0.001). Considering menopausal status, the distribution of hormone receptors in premenopausal patients was ER(+)/PgR(+) 63.76%, ER(−)/PgR(−) 23.49%, ER(+)/PgR(−) 10.74%, and ER(−)/PgR(+) 2.01%, respectively, while in postmenopausal women, this distribution was observed to be 74.29%, 23.81%, 1.90% and 0.00% in the same order. The correlation between ER and PgR levels was weak during the premenopausal period and strong during the postmenopausal period.

### 3.3. Molecular Classification Analysis Results

**Figure 1:**
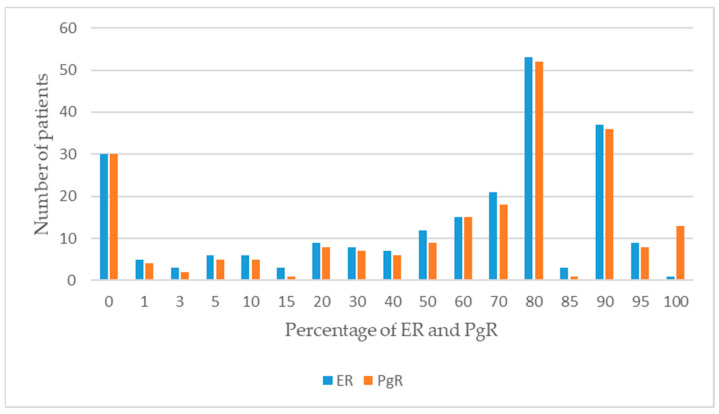
Distribution of patients according to hormone receptors.

**Figure 2:**
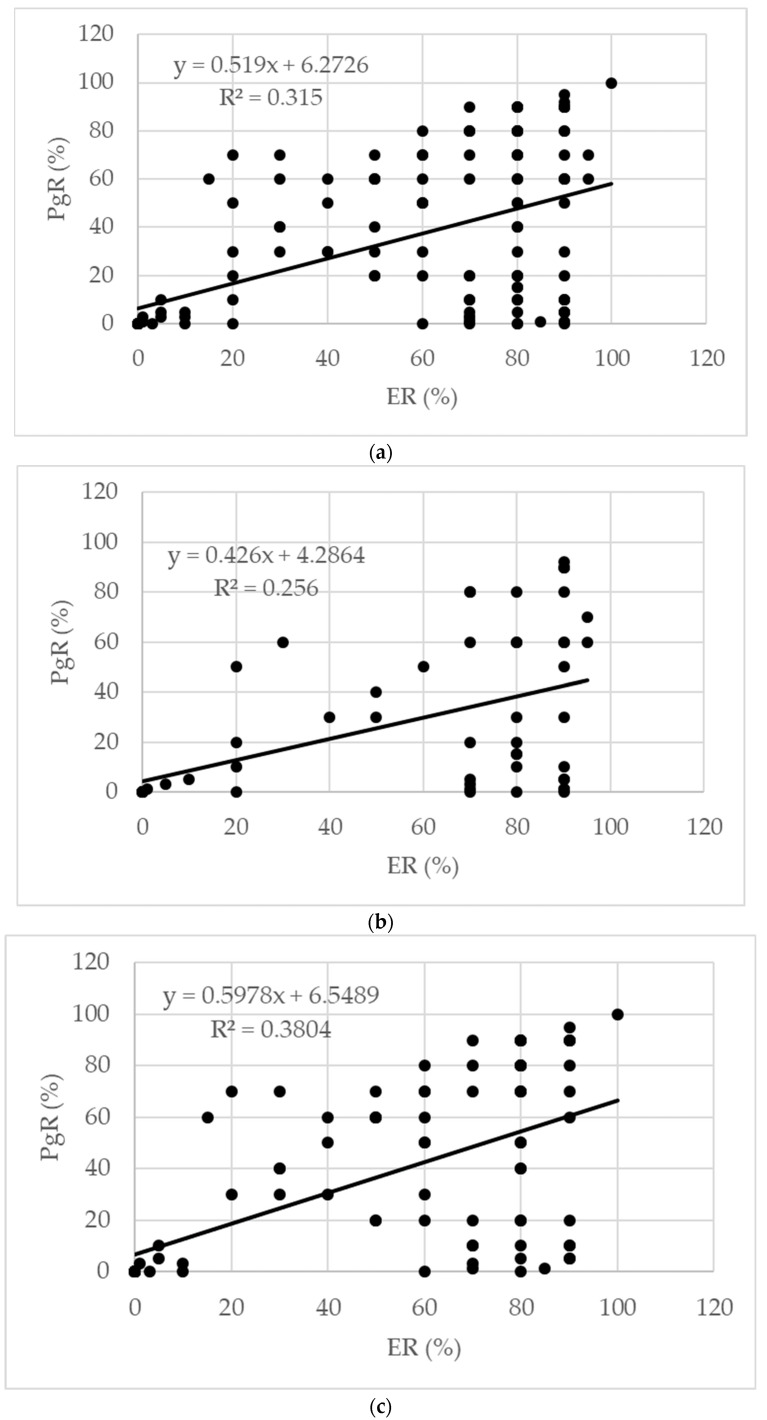
Correlations between estrogen and progesterone receptors in (**a**) all, (**b**) postmenopausal BCa, and (**c**) premenopausal BCa patients.

When the patients were classified according to BCa subtypes, the percentages of patients in luminal A, luminal B-HER2(−), luminal B-HER2(+), HER2 positive, and triple-negative subgroups among all patients were 36.61%, %11.42, 25.59%, 9.06%, and 17.32%, respectively. Among premenopausal patients, the percentages of patients in BCa subtypes were determined to be 34.90%, 8.05%, 26.17%, 10.74%, and 20.13%, respectively, while in the postmenopausal group, these values were 39.05%, 16.19%, 24.76%, 6.67%, and 13.33%, respectively. The clinicopathological characteristics of the patients are presented in Table 2. The distribution of the patients according to their hormone receptors is given in Figure 1. Correlations between the hormone receptors in the patients are presented in Figure 2.

### 3.4. Somatic Mutation Profiles

**Figure 3:**
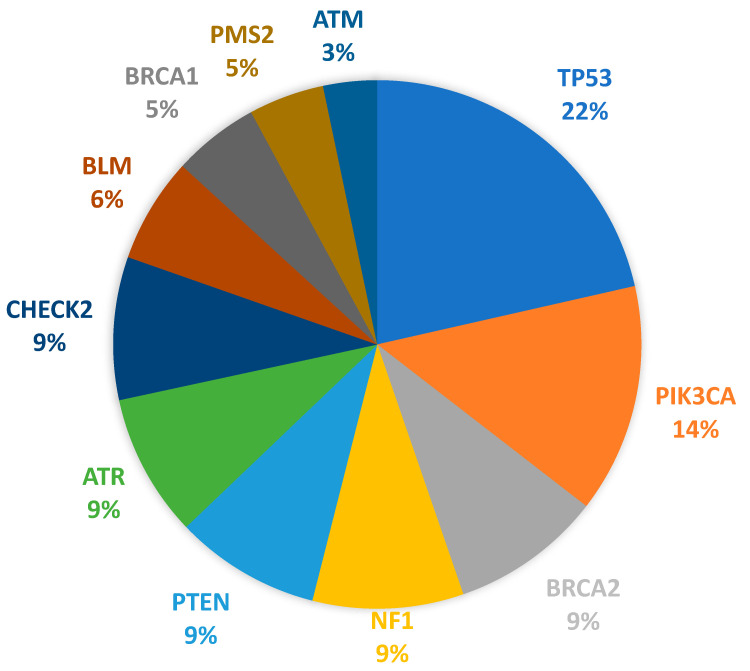
Pathogenic gene variants in BCa patients.

Somatic mutations were detected in a total of 94.09% (*n* = 239/254) of the tested patients. Among the 62 genes covered, including frequently mutated genes, a total of 1366 pathogenic, likely pathogenic, and variants of uncertain significance were identified. Among these variants, 296 variants were of uncertain significance. The most frequently mutated gene among pathogenic variants was *TP53*, with 35 different variants observed in 130 patients (51.18%). This was followed by *PIK3CA* with 14 different variants observed in 85 patients (33.46%), *BRCA2* with 10 different variants and NF1 with 2 different variants both observed in 56 patients (22.05%), *PTEN* with 8 different variants observed in 54 patients (21.26%), and *ATR* with 4 different variants and *CHEK2* with 7 different variants observed in 53 patients (20.87%). Among the less frequently mutated genes, *BLM, BRCA1, PMS2*, and *ATM* variants were observed in 39 (15.35%), 32 (12.60%), 28 (11.02%), and 20 (7.87%) patients, respectively (Figure 3). The pathogenic variants observed in the ten most commonly mutated genes are shown in Table 3.

The distinct mutational characteristics for each molecular subtype of BCa were listed in Table 1. *TP53* mutations were detected in 54.55% of the HER2 positive subtype (*n* = 12/22), 31.03% of luminal B-HER2(−) (*n* = 9/29), 17.07% of triple-negative (*n* = 7/41), 16.13% of luminal A (*n* = 15/93), and 10.77% of luminal B-HER2(+) subtypes (*n* = 7/65). The mutations in the *PIK3CA* gene were detected in 22.72% of HER2 positive subtype (*n* = 5/22) followed by luminal A (*n* = 18/93, 19.35%), luminal B-HER2(−) (*n* = 1/29, 3.45%), luminal B-HER2(+) (*n* = 9/65, 13.85%), and triple-negative (*n* = 3/41, 7.32%). The luminal A subtype presented the *BRCA2* mutations the most by 9.67% (*n* = 9/93) among other subtypes, while the luminal B-HER2(+) subtype for the *NF1* mutations by 12.31% (*n* = 8/65).

**Figure 4:**
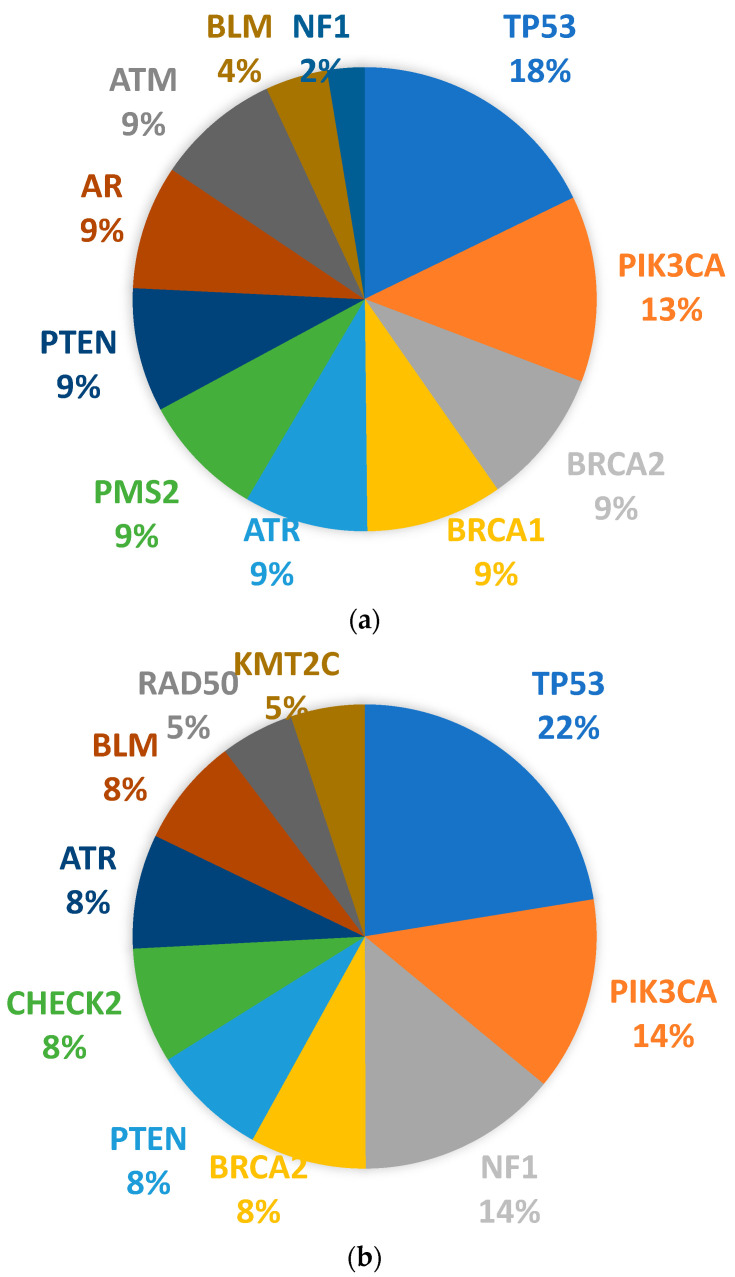
Pathogenic variants in BCa according to menopausal status. (**a**) postmenopausal BCa, (**b**) premenopausal BCa.

In premenopausal BCa patients, *TP53, PIK3CA, NF1, BRCA2, PTEN, CHEK2, ATR, BLM, RAD50,* and *KMT2C* somatic mutations were observed more frequently, while in postmenopausal BCa patients, *TP53, PIK3CA, BRCA2, BRCA1, ATR, PMS2, PTEN, AR, ATM, BLM,* and *NF1* mutations were more commonly detected. The top 10 genes most frequently mutated according to menopausal status are shown in Figure 4. Mutations were more commonly observed in the luminal B-HER2(+) subtype in both premenopausal and postmenopausal patients. The mutation burden was higher in postmenopausal BCa patients.

## 4. Discussion

Tenea-Cojan et al. (2016) reported that the rate of non-special type invasive breast carcinoma was 63.37%, followed by invasive lobular carcinoma at 10.56% [8]. Consistent with these findings, the majority of tumors in our study consisted of invasive ductal carcinoma at 85.04%, while 10.24% were invasive lobular carcinoma.

When evaluating the mutual relationships of hormone receptors obtained in this study, it is observed that the ER(+)/PgR(+) profile is the most prevalent at 68.11%, followed by ER(−)/PgR(−) at 23.62%, ER(+)/PgR(−) at 7.09%, and ER(−)/PgR(+) at 1.81%, which is consistent with the findings of Chu et al. (2001). These researchers also reported in their study that the majority of cases had the ER(+)/PgR(+) profile (63.9%) [9,10]. Additionally, a robust positive correlation was present between ER and PgR in our study (*p* < 0.001).

In the last decade, studies on the molecular biology of BCa and the data obtained have enabled the molecular classification of the disease. The integration of molecular markers into conventional BCa classification systems has allowed for more effective treatment guidance and the exploration of potential treatment targets [11]. It has been noted that besides traditional prognostic tools, the immunohistochemical molecular classification of BCa may help detect patients with varying recurrence risks and provide insights into cancer treatment [12]. Especially, the accurate classification of Luminal A and Luminal B subtypes is emphasized to be of extreme importance in determining treatment [13]. In a prevalence study of molecular subtypes and hormone receptors conducted by Pandit et al. (2020), it was found that out of 2062 patients examined, the Luminal A subtype was observed in 37%, Luminal B subtype in 7.6%, basal-like subtype in 26%, and HER2-enriched subtype in 11.1% of the patients. It was observed that the incidence of the Luminal A subtype increased with age, while the incidence of the basal-like subtype was highest in patients under 30 years old [14]. In a study by Özmen et al. (2014), which analyzed the subtypes of tumors in Turkey, it was shown that 62% were luminal A subtype, followed by luminal B (15%), triple-negative (15%), and HER2 positive (8.5%) subtypes [15]. In our study, it was found that 36.61% of the patients were Luminal A, 11.42% were luminal B-HER2(−), 25.59% were luminal B-HER2(+), 9.06% were HER2 positive, and 17.32% were triple-negative subtypes. We believe that the lower percentages of molecular subtypes observed in our study compared to this national data [15] may be partially associated with the relatively small sample size in our study. In our study, the Luminal A molecular subtype was the most common (36.61%) among all BCa cases. Luminal A BCa subtype is more commonly encountered in postmenopausal women [16], and our study also found that Luminal A is the more prevalent molecular subtype in postmenopausal women. However, despite the increasing knowledge about prognostic factors, there is no comparative data on molecular subtypes and overall survival prognostic factors among all premenopausal and postmenopausal women with BCa in the entire population. Previous studies, such as the Carolina BCa Study, have reported a higher prevalence of basal-like breast tumors in premenopausal BCa patients in comparison to postmenopausal patients [17], consistent with the current study.

Fluctuating hormone levels before and after menopause likely influence the gene expression patterns as detected between premenopausal and postmenopausal BCa patients [18]. These findings revealed that certain genes may function in a menopausal status-dependent fashion. It was reported that somatic mutations in *TP53* were observed in 47.6% of premenopausal BCa samples, while 38.1% exhibited mutations in *PIK3CA* [19]. Similar results were also obtained in a study conducted on premenopausal BCa patients of Latin American descent, in which *TP53* and *PIK3CA* emerged as the two most commonly mutated genes [20]. It is also indicated in the same report that a clinical correlation was found between somatic *TP53* mutations and the HER2 positive molecular subtype. The same association was also reported in a premenopausal cohort [21] and by another study that did not focus on menopausal status [22]. The potential of *TP53* to upregulate the *HER2* expression was the reason for this association [23]. Nagy et al. (2021) showed in their study that the *PIK3CA* somatic mutation frequencies were 37% and 17% in postmenopausal and young patients, respectively, E454A being the most prevalent *PIK3CA* mutation [24]. In the current study, *TP53* and *PIK3CA* were found to be among the genes with the highest mutation frequencies in BCa cases, suggesting their pivotal roles in carcinogenesis. Also, the *TP53* and *PIK3CA* mutations were mostly observed in the HER2 positive molecular subtype, in line with the literature. Studies list *PIK3CA* mutations among the less frequent mutations in younger women than in older ones. In fact, for advanced BCa, *PIK3CA* mutations are regarded as poor prognostic factors when compared against early BCa [25,26]. On the contrary, the current study detected *PIK3CA* mutations more frequently in younger patients in the premenopausal period. In this sense, the correlation of PI3K pathway activity level with age and/or menopausal status may require further research.

The most observed mutations in *PIK3CA* tend to concentrate in the codons E542K (c.1624G>A) and E545K (c.1633G>A) in exon 9, which is part of the helical domain, and also in the codon H1047R (c.3140A>G) in exon 20. Exon 9 resides in helical domain while exon 20 in the kinase domain [27,28]. One report indicated E545A as the most common mutation observed in *PIK3CA* that has intermediate oncogenic potential [27]. A similar finding was also reported by two other studies conducted in Singapore and Peru on subjects with BCa [29,30]. Today, E454A is a recognized mutation with its own specific method for detection [31]. On the other hand, other point mutations in *PIK3CA* were also reported as being hotspots such as E542K, E545K, and H1047R in a study recruiting Brazilian BCa patients followed by H1047L and S553FS mutations [32]. H1047L is known to have high oncogenic potential [27]. Additionally, the S553FS frameshift mutation may abolish the proto-oncogenic potential of *PIK3CA.* Furthermore, nonsense mutations in *PIK3CA* were identified in tumor tissues of both older and younger patients, potentially counteracting the proto-oncogenic effects of *PIK3CA*. However, contrasting findings have been reported in another study, indicating that nonsense mutations are not as commonly observed in *PIK3CA* [32]. In our study, the most frequently detected *PIK3CA* variants were E545K, H10477R, E542K, and H1047L, consistent with the literature, and among these variants, E545K and E542K located in the helix domain were observed in postmenopausal BCa cases, and H10477R and H1047L located in the kinase domain were observed in premenopausal BCa cases. Additionally, in our study, other than these hotspot mutations, missense and nonsense type single nucleotide variants were detected in exons 2, 3, 5, 7, 10, 14, 18, 19, and 21. These variants of *PIK3CA* may be related to a higher BCa risk.

Calculating the potential of germline *BRCA* mutations requires the family history of patients for breast and ovarian cancers and also the age of onset [33]. Germline mutations in *BRCA1* and *BRCA2* account for approximately 30% of hereditary Bcas globally [34]. A large study conducted in Brazil with 1554 Bca patients found that 9.84% of patients were carriers of *BRCA1* or *BRCA2* mutations regardless of their ages [35]. Other studies have reported higher frequencies of *BRCA* mutations in young Brazilian Bca patients up to the age of 35 (ranging from 15% to 22%) [35,36,37]. In high-risk Saudi patients, the rates of *BRCA1* and *BRCA2* mutations in Bca tumors were reported to be 12.9% [38], while another Saudi study reported higher somatic mutation rates (30.18% for *BRCA1* and 37.7% for *BRCA2*) [34]. However, there is limited data in the literature specifically on somatic mutations in Bca patients related to *BRCA1* and *BRCA2* genes. In our study, we did not examine germline mutations in these genes, and similar to the literature, the rates of somatic mutations in *BRCA1* and *BRCA2* were observed to be 12.06% and 22.05%, respectively.

The remaining cases exhibited vari”ble ’utations in different combinations (*NF1, PTEN, ATR, CHEK2, PMS2,* and *ATM*), as previously reported, showing lower frequencies compared to a set of *TP53* and *PIK3CA* mutations [39]. However, conversely, high-frequency variants were detected in the *BLM* gene, which was reported to rarely occur in Bca [34,40]. This observation emphasizes the complexity of oncogenic interactions among genes carrying mutations, highlighting that these interactions are not straightforward or linear processes. Instead, they are shaped by intricate sequences comprising tightly interconnected molecular networks and pathways.

The *NF1* gene has been shown to be a causative agent of breast cancer, with somatic mutations reported in 27.7% of all breast carcinomas [41,42]. Previous studies have suggested that a mutation in the *NF1* gene may result in or predispose cells to mutations in other genes on the same chromosome [43]. The *NF1* gene and the *BRCA1* gene are both located approximately 20 centi-Morgan (cM) apart on chromosome 17, and an interaction between these two genes has been suggested [44,45]. However, the risk of breast cancer in patients found to have a variant in the *NF1* gene without any clinical evidence is unclear. In our study, *NF1* mutations were detected in 22.05% of all Bca cases, similar to the literature. Especially in our study results, the presence of *NF1* mutations together with *BRCA1* mutations in women with postmenopausal Bca may bring up the possibility of an interaction between the two genes.

This report adds to the limited body of studies providing insights into the frequency of somatic mutations in both premenopausal and postmenopausal Bca cases, utilizing robust NGS technology.

## 5. Conclusions

In conclusion, our study revealed that 94.09% of both premenopausal and postmenopausal Bca cases harbored somatic mutations in established cancer susceptibility genes. These findings contribute to a deeper understanding of the underlying causes of Bca with respect to menopausal status. Given the high prevalence of genetic mutations identified, genetic testing holds promise not only for informing treatment decisions for both premenopausal and postmenopausal Bca patients but also for shaping future prevention and management strategies to mitigate the risk of secondary malignancies in patients.

## Figures and Tables

**Table 1:** The gene list in the QIAseq Human Breast Cancer NGS Panel.

*ACVR1B*	*BMPR1A*	*CDKN2A*	*ERCC4*	*GATA3*	*MDM2*	*NBN*	*PMS1*	*SEPT9*	*XRRC2*
*AKT1*	*BRCA1*	*CHEK2*	*ESR1*	*GEN1*	*MED12*	*NCOR1*	*PMS2*	*SMAD4*	*XRRC3*
*APC*	*BRCA2*	*CSMD1*	*EXT2*	*HERC1*	*MEN1*	*NEK2*	*PPM1L*	*SMARCA4*	*ZBED4*
*AR*	*BRIP1*	*CTNNB1*	*EXOC2*	*HOXB13*	*MLH1*	*NF1*	*PTEN*	*STK11*	*-*
*ATM*	*CASP8*	*DIRAS3*	*FAM175A*	*IRAK4*	*MRE11A*	*PALB2*	*PTGFR*	*SYNE1*	*-*
*ATR*	*CBFB*	*EGFR*	*FBXO32*	*ITCH*	*MSH2*	*PALLD*	*RAD50*	*TGFB1*	*-*
*AXIN2*	*CCND1*	*EP300*	*FANCC*	*KMT2C*	*MSH6*	*PBRM1*	*RAD51C*	*TP53*	*-*
*BAP1*	*CDH1*	*EPCAM*	*FBXO32*	*KRAS*	*MUC16*	*PCGF2*	*RAD51D*	*TRAF5*	*-*
*BARD1*	*CDK4*	*ERBB2*	*FGFR1*	*MAP2K4*	*MUTYH*	*PIK3CA*	*RB1*	*VHL*	*-*
*BLM*	*CDK6*	*ERBB3*	*FGFR2*	*MAP3K1*	*MYC*	*PIK3R1*	*RET*	*WEE1*	*-*

**Table 2:** Clinical and pathological features of BCa patients according to menopausal status.

Features	Postmenopausal	Premenopausal	*p* Value
	*n* = 105	*n* = 149	
Age at diagnosis, median (range), years	59.54 ± 9.01	42.11 ± 5.51	0.000 *
Special Histopathology Subtypes n (%)			
Invasive ductal carcinoma	87 (82.86)	129 (86.58)	0.186
Invasive lobular carcinoma	10 (9.52)	16 (10.74)	
Other special types of carcinomas	8 (7.62)	4 (2.68)	
Hormone receptor status			
ER(+)/PgR(+)	78 (74.29)	95 (63.76)	0.008 *
ER(−)/PgR(−)	25 (23.81)	35 (23.49)	
ER(+)/PgR(−)	2 (1.90)	16 (10.74)	
ER(−)/PgR(+)	0 (0.00)	3 (2.01)	
Tumor Subtype n (%)			
Luminal A	41 (39.05)	52 (34.90)	0.154
Luminal B-HER2 (+)	26 (24.76)	39 (26.17)	
Luminal B-HER2 (−)	17 (16.19)	12 (8.05)	
HER2 positive	7 (6.67)	16 (10.74)	
Triple Negative	14 (13.33)	30 (20.13)	

*: Significant *p* < 0.05.

**Table 3:** Pathogenic variants observed in the 10 most commonly mutated genes in BCa patients.

Genes	Mutations
*TP53*	
*Frameshift variants*	Exon 4 c.267delC Exon 5 c.389delT Exon 5 c.390_426delCAACAAGATGTTTT Exon 5 p.481delG Exon 7 c.737_740delTGAA Exon 7 c.754delC Exon 7 c.774dupA Exon 7 c.780delC Exon 8 c.803-805delACA Exon 10 c.1024delC Exon 13 c.323_329dupGTTTCCG Exon 13 c.576dupG Exon 4 c.158G>A Exon 4 c.372C>A Exon 5 c.497C>G Exon 5 c.499C>T Exon 8 c.916C>T Exon 8 c.1024C>T Exon 20 c.1024C>T Exon 5 c.469G>T Exon 5 c.524G>A Exon 5 c.730G>T Exon 6 c.584T>C Exon 6 c.659A>G Exon 7 c.524G>A Exon 7 c.742C>T Exon 7 c.743G>A Exon 8 c.818G>A Exon 8 c.853G>A Exon 10 c.329G>C Exon 11 c.818G>A Exon 13 c.856G>A Exon 6 c.920-1G>T Exon 9 c.920-2A>T Exon 11 c.994-2A>G











*Nonsense variants*	






*Missense variants*	












*Splice acceptor variants*	
*PIK3CA*	
*Nonsense variants*	Exon 2 c.277C>T
	Exon 3 c.353G>A
	Exon 5 c.1035T>A
	Exon 7 c.3127A>G
	Exon 9 c.1624G>A
	Exon 9 c.1633G>A
	Exon 9 c.1634A>C
	Exon 10 c.3127A>G
	Exon 14 c.2176G>A
	Exon 18 c.1637A>G
	Exon 19 c.2702G>T
	Exon 21 c.23145G>C
*Missense variants*	Exon 20 c.3140A>G
	Exon 20 c.3140 A>T
*BRCA2*	Exon 7 c.3847_3848delGT Exon 10 c.1813delA Exon 11 c.3539delA Exon 11 c.5073delA Exon 18 c.8331+1delG Exon 23 c.9097delA Exon 11 c.4440T>G Exon 18 c.1103C>G Exon 20 c.8504C>G Exon 25 c.9382C>T
*Frameshift variants*	





*Nonsense variants*	
*NF1*	
*Nonsense variant*	Exon 13 c.1400C>T
*Intron variant*	Exon 19 c.2325+3A>G
*PTEN*	
*Frameshift variants*	Exon 8 c.802-2delA
	Exon 15 c.692_708delCCACACGACGGGAAGAC
*Nonsense variants*	Exon 8 c.697C>T
	Exon 8 c.1003C>T
*Missense variants*	Exon 5 c.407G>A
	Exon 10 c.397G>A
	Exon 18 c.389G>A
*Splice donor variant*	Exon 4 c.253+1G>C
*ATR*	
*Frameshift variants*	Exon 10 c.2320delA
	Exon 10 c.2319_2320delAA
	Exon 10 c.2320duplA
*Nonsense variant*	Exon 6 c.3547C>T
*CHEK2*	Exon 8 c.1450_1451delCCinsT Exon 12 c.1361G>A Exon 6 c.737A>G Exon 10 c.1427C>T Exon 10 c.1556C>T Exon 12 c.1312G>T Exon 14 c.1556C>T
*Frameshift variant*	
*Nonsense variant*	
*Missense variants*	
*BLM*	
*Frameshift variants*	Exon 7 c.1544delA
	Exon 7 c.2320delA
*Nonsense variant*	Exon 8 c.1642C>T
*BRCA1*	
*Frameshift variants*	Exon 3 c.3794delA
	Exon 10 c.1961delA
	Exon 10 c.3333delA
	Exon 10 c.3770_3771delAG
	Exon 16 c.5030_5033delCTAA
	Exon 2 c.66dupA
	Exon 19 c.5266dupC
*Splice donor variant*	Exon 3 c.134+2T>C
*Splice acceptor variant*	Exon 4 c.135-2A >G
*PMS2*	
*Frameshift variants*	Exon 11 c.1239delA
	Exon 11 c.2165delA
*ATM*	
*Frameshift variant*	Exon 6 c.640delT
*Nonsense variant*	Exon 7 c.742C>T
*Missense variants*	Exon 8 c.1009C>T
	Exon 17 c.2572T>C
	Exon 22 c.3161C>G
	Exon 50 c.7463G>A

## Data Availability

Additional data are available upon request.

## Supplementary Materials

The following supporting information can be downloaded at: https://www.mdpi.com/article/10.3390/jpm14040434/s1, Figure S1: (a) HER2+ FISH image; (b) HER2- FISH image.
