# Supplementary figures and images for: Variation Analysis in Premenopausal and Postmenopausal Breast Cancer Cases
Source: J Pers Med. 2024 Apr 20;14(4):434. doi: 10.3390/jpm14040434 (PMC11051104; doi:10.3390/jpm14040434)

Supplementary materials

Figure S1. (a) HER2+ FISH image (b) HER2- FISH image

S1a.

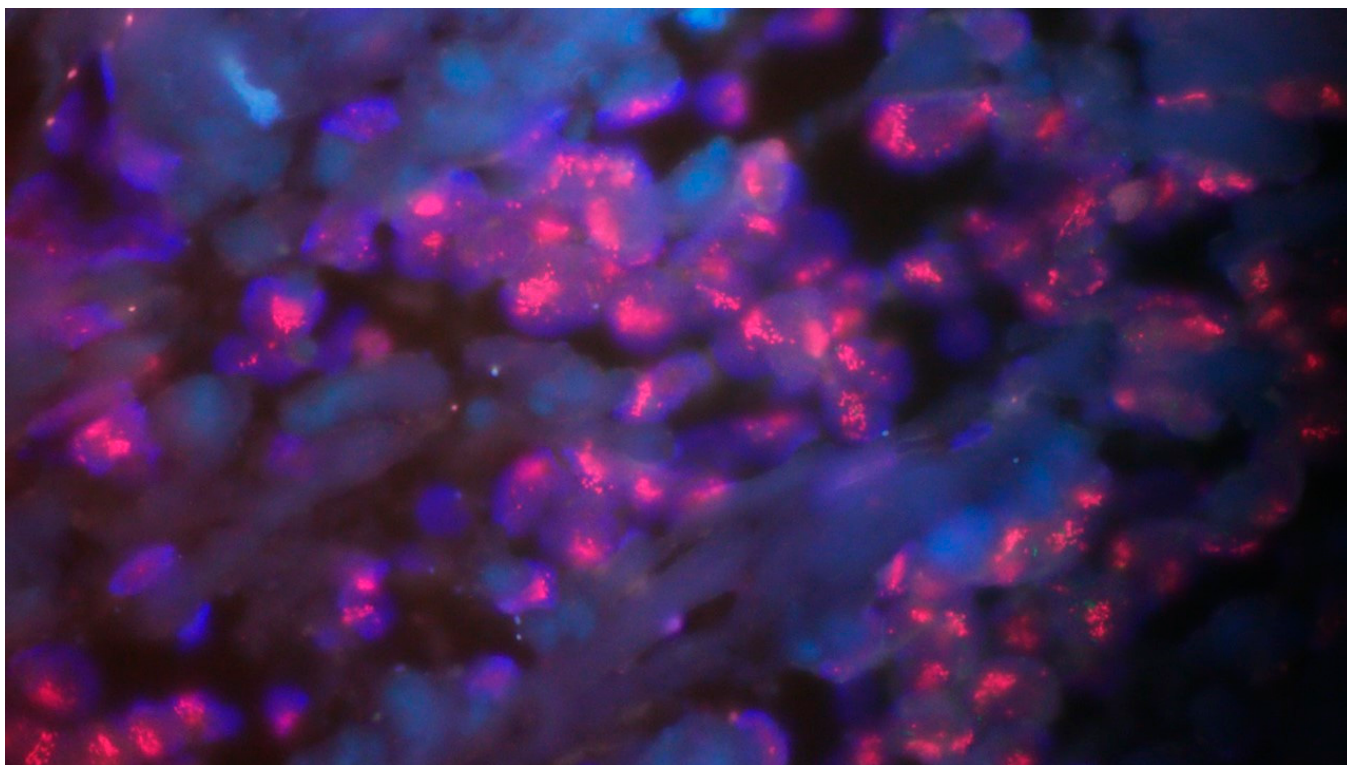

S1b.

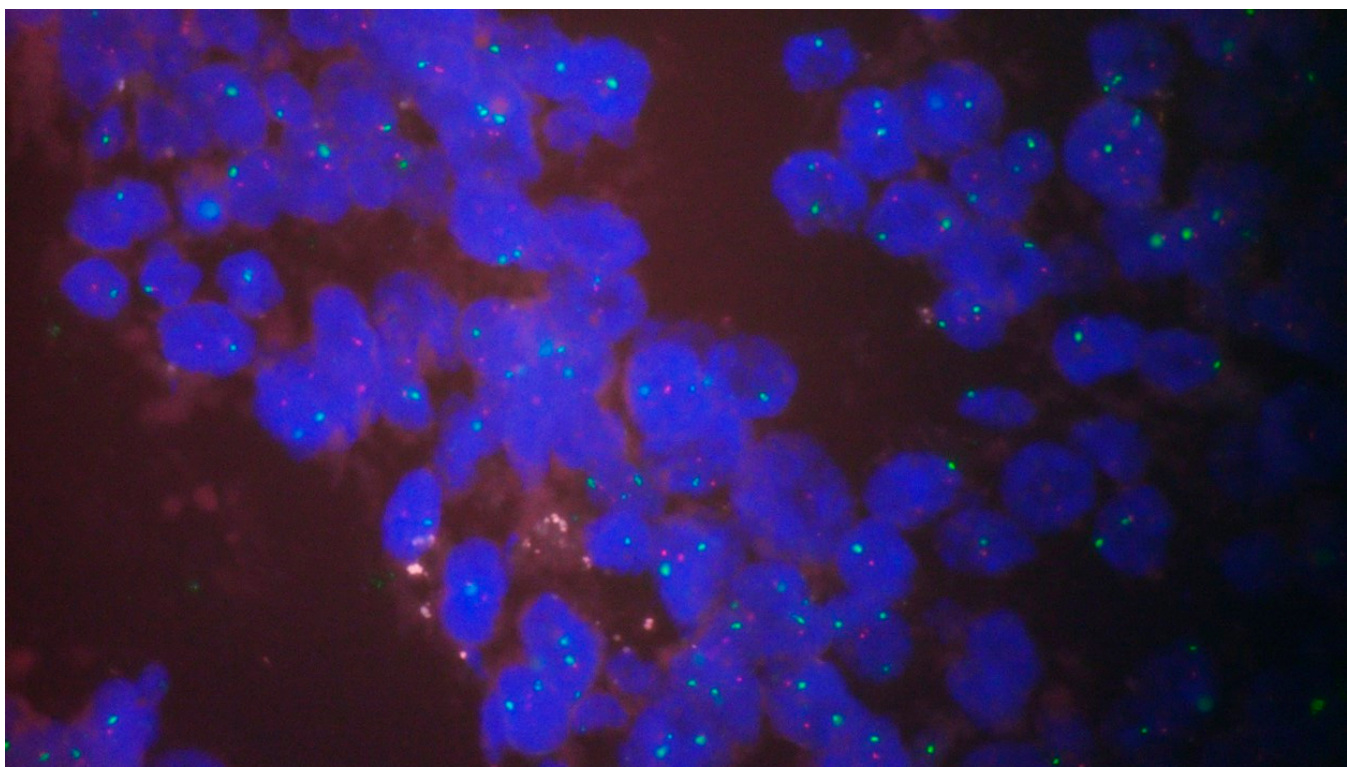

Supplement: Supplementary file 1 [file jpm-14-00434-s001.zip › jpm-2952657-supplementary.pdf]
